# Model Analysis of Robotic Soft Arms including External Force Effects

**DOI:** 10.3390/mi13030350

**Published:** 2022-02-23

**Authors:** Zhi Chen, Zhong Liu, Xingguo Han

**Affiliations:** School of Mechanical Engineering, Guilin University of Aerospace Technology, Guilin 541004, China; chenzhi@guat.edu.cn (Z.C.); liuzhong678@163.com (Z.L.)

**Keywords:** robotic soft arm, constant curvature model, Euler–Bernoulli beam theory, cable-driven, external force

## Abstract

Because robotic soft arms have a high power-to-weight ratio, low cost, and ease of manufacturability, increasing numbers of researchers have begun to focus on their characteristics in recent years. However, many urgent problems remain to be resolved. For example, soft arms are made of hyperelastic material, making it difficult to obtain accurate model predictions of the soft arm shape. This paper proposes a new modeling method for soft arms, combining the constant curvature model with Euler–Bernoulli beam theory. By combining these two modeling methods, we can quickly solve for the soft arm deformation under the action of an external force. This paper also presents an experimental platform based on a cable-driven soft arm to verify the validity of the proposed model. We carried out model verification experiments to test for different external effects. Experimental results show that the maximum error of our proposed soft arm deformation model is between 2.86% and 8.75%, demonstrating its effectiveness.

## 1. Introduction

Soft arms play an increasingly important role in robotics because of their flexibility, adaptability to complex environments, and safety in human–computer interactions [1,2,3,4]. However, soft arms are made of hyperelastic material, with drawbacks, such as strong nonlinearity and considerable deformation. These deficiencies ultimately make it difficult to model the soft arm. This difficulty, combined with its susceptibility to interference from external forces, makes it challenging to predict the soft arm shape.

Increasing numbers of researchers have begun focusing on soft arm modeling methods [5,6,7]. Existing methods can be classified into two categories: (1) methods based on constant curvature; and (2) methods based on variable curvature.

Thien-Dang et al. [8] proposed a novel tendon-driven continuum robot with extensible sections based on the constant curvature assumption. The continuum robot is expected to be used in medical operations. Zheng Li et al. [9] presented a bio-inspired wire-driven multisection flexible robot and developed kinematics and workspace models based on the piece-wise constant curvature assumption. Gaurav Singh et al. [10,11,12] analyzed the deformation of fiber-reinforced elastomeric actuators based on the constrained maximization formulation and virtual work principle. The model accurately captured the deformed shape for a soft arm with any general fiber angle orientation. Polygerinos et al. [13] comprehensively analyzed the working principles of the soft arm, combining finite element analysis and geometric modeling methods to verify the soft arm deformation behavior through simulation and experimental verification. Fionnuala Connolly et al. [14] believe that the mathematical modeling of soft actuators is still in its infancy, and proposed a design strategy that takes motion trajectory as input. This strategy can be used to guide the soft arm design. Joan et al. [15] designed a soft three-modular-section robot driven by a shape memory alloy inspired by the motion generated by the bowel. Inspired by the elephant trunk, Guan et al. [16] analyzed and discussed the soft arm’s axial, bending, and torsion characteristics based on a coupled constant curvature and torsion kinematics model. Gong et al. [17] designed and fabricated a soft manipulator with an opposite-bending-and-stretching structure. A simple and efficient kinematics method was proposed for controlling the spatial location and trajectory of the soft manipulator’s end effector. The research uses a camera to track the soft arm’s posture and continuously adjusts the input air pressure based on this information. The most significant advantage of the constant curvature-based modeling method is its fast solution speed. The lack of complicated calculations allows the soft arm shape to be obtained in real-time. However, models based on the constant curvature assumption can only be used under ideal conditions. In a complex and changeable environment, soft arm modeling based on constant curvature cannot meet actual needs, especially when considering the interference of external forces on the soft arm shape.

Xu et al. [18] proposed a dynamic soft arm underwater environment model based on Kane’s theory [19]. The model considers the behavior of the soft arm’s shape under other external force conditions, such as complex fluid mechanics. Renda et al. [20,21,22] conducted a series of analyses on the force of the soft arm based on Cosserat rod theory [23,24] and in their work, statics and dynamics modeling and analysis of the soft arm are carried out. The shear strain factor is also considered in the dynamic modeling analysis. Huang et al. [25] proposed variable curvature kinematic modeling of a soft arm based on the absolute nodal coordinate formulation. This model can calculate the influence of external loads on the soft arm shape. Naveen et al. [26] proposed a modeling method that combines geometric modeling and Cosserat rod theory. The model focuses on gravity’s influence on the shape of the soft arm. Rucker et al. [27] proposed a modeling method that includes the soft arm’s statics and dynamics and also considers the influence of external forces. The modeling methods mentioned above all account for the influence of external forces, and the proposed model can also effectively predict the shape of the soft arm. Finite element modeling [28,29,30,31] provides an efficient solution due to the strong non-linearities and we can also use this method to predict performance and optimize soft actuator designs. Grazioso et al. [32] proposed the deformation space formulation for soft robots dynamics, developed using a finite element approach. They combined the finite element method with Cosserat rod theory to model soft robotic arms subject to external forces and three-dimensional motions. However, these methods mentioned above also have disadvantages, such as complex calculations, difficult implementation, and possible non-convergence of the desired results.

This paper proposes a new method combining the constant curvature model with Euler–Bernoulli beam theory to predict the soft arm deformation quickly and effectively. The constant curvature model can quickly provide the curvature of the current soft arm. The Euler–Bernoulli beam theory, based on the constant curvature model, can account for the interference of external forces on soft arm deformation. Combining these two models permits effective prediction of soft arm deformation under external force conditions.

This paper is organized as follows. The model derivation method based on constant curvature and the Euler–Bernoulli beam equation is provided in Section 2. We introduce the experimental platform construction and preliminary preparations for model verification in Section 3. In Section 4, the experiments carried out to verify the model’s effectiveness are presented along with the model verification results. Finally, we summarize relevant conclusions and discuss plans for future work in Section 5.

## 2. Derivation of Soft Arm Model

This section derives the constant curvature modeling method of the soft arm and the Euler–Bernoulli beam theory modeling method.

### 2.1. Constant Curvature Modeling

In this paper, we use the length change of the cable on both sides to measure the degree of bending of the soft arm. It is worth noting that we assume that the soft arm itself is incompressible.

**Figure 1:**
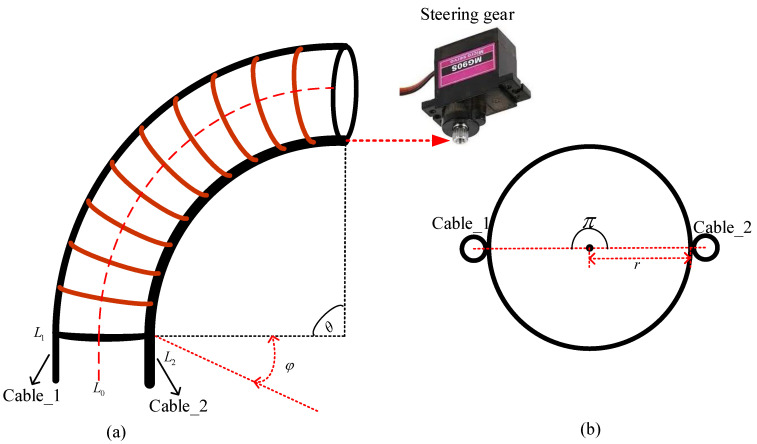
(**a**) Diagram of the bending of the soft arm; (**b**) Cross-sectional schematic diagram of the soft arm and the arrangement of the cable.

The bending state of the soft arm can be achieved by controlling the contraction state of the two cables with the steering gear, as shown in Figure 1a, where θ is the bending angle, φ is the torsion angle, and $L_{1},L_{2}$ represent the cable length on either side of the soft arm. The backbone configuration of the soft arm is denoted by $L_{0}$.

(1) $$\Delta L_{1}=L_{1}-L_{0},\Delta L_{2}=L_{2}-L_{0}.$$

Because the two cables are arranged 180 degrees apart, and the inner radius of the soft arm is set to r, Equation (1) can be further derived as:

(2) $$\begin{array}{l} \Delta L_{1}=r\theta \cos\varphi ,\\ \Delta L_{2}=r\theta \cos\left(\varphi -\pi \right)=-r\theta \cos\varphi \end{array}$$

The following relationship can be obtained from Equation (2):

(3) $$\Delta L_{1}+\Delta L_{2}=0.$$

In addition, because the soft arm can bend in only one plane in this study, the torsion angle φ equals zero resulting in the following relationships:

(4) $$\begin{array}{l} \Delta L_{1}=r\theta \cos\varphi =r\theta ,\\ \Delta L_{2}=r\theta \cos\left(\varphi -\pi \right)=-r\theta \cos\varphi =-r\theta \end{array}$$

**Figure 2:**
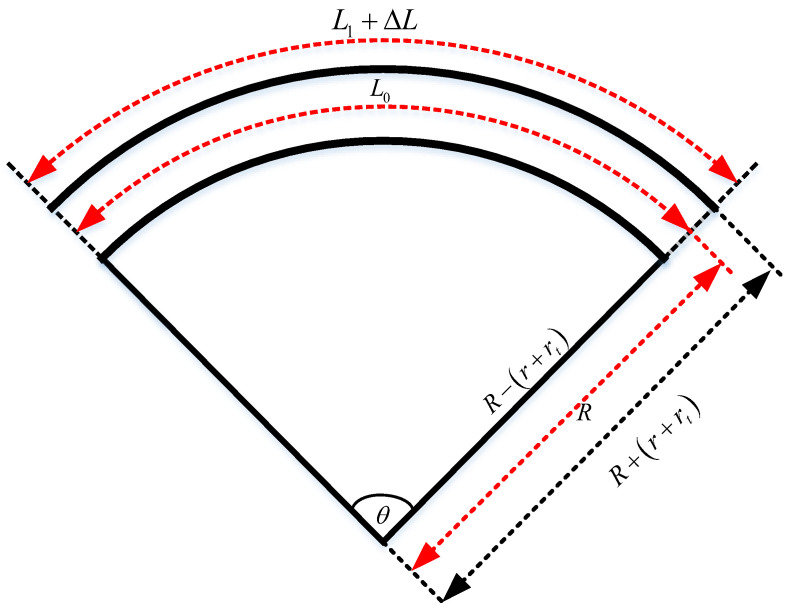
Diagram of the bending of the soft arm.

Using Figure 2, we can obtain the relationship between the change in cable length and the curvature, or the radius of curvature, of the soft arm, as given in Equation (5), where $r_{t}$ is the soft arm wall thickness. R and k denote the radius of curvature and curvature, respectively.

(5) $$\frac{R+r+r_{t}}{R}=\frac{L_{0}+\Delta L}{L_{0}}\to R=\frac{L_{0}\left(r+r_{t}\right)}{\Delta L}=\frac{1}{k}.$$

### 2.2. Derivation of Euler–Bernoulli Beam Model

**Figure 3:**
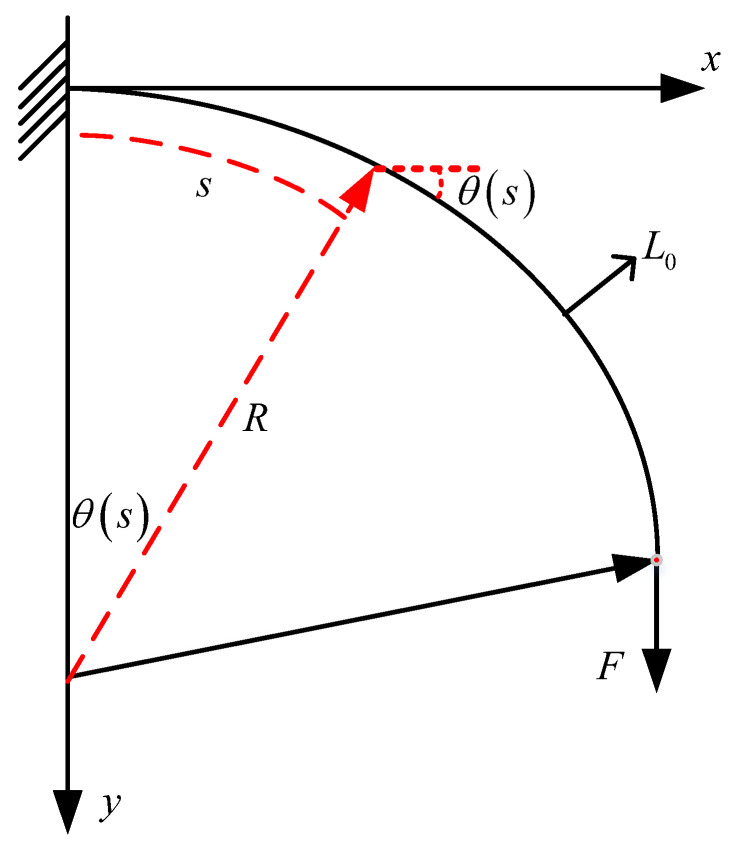
Diagram of the soft arm subjected to an external vertical force at one end.

For the force application situation of the soft arm depicted in Figure 3, we can obtain the following geometric relationships:

(6) $$\theta \left(s\right)=\frac{s}{R},\theta^{\prime }\left(s\right)=\frac{1}{R}=k.$$

In addition, because the constant initial curvature is $\frac{1}{R}$, $\theta^{\prime }\left(s\right)$ represents the first derivative with respect to s, and thus, the derivative of k equals zero. This article addresses two force modes of the soft arm:

(1) a vertical force on the end of the soft arm;
(2) an external force applied to any position on the soft arm.

For the first condition, shown in Figure 3, we can use the following Euler–Bernoulli beam equation [33,34,35,36]:

(7) $$EI\left(\frac{d^{2}\theta \left(s\right)}{ds^{2}}-\frac{dk_{0}}{ds}\right)+F\cos\theta \left(s\right)=0,$$

where *E* is the beam elastic modulus, and *I* is the beam moment of inertia.

Equation (7) should satisfy the following boundary conditions:

(8) $$\theta \left(0\right)=0,\theta^{\prime }\left(L\right)=k.$$

After θ is obtained by solving the ordinary differential equation, the following equations can be used to solve for the final shape of the soft arm.

(9) $$\begin{array}{l} x\left(s\right)=\int_{s}^{L}\cos\left(\theta \left(s\right)\right)ds\\ y\left(s\right)=\int_{s}^{L}\sin\left(\theta \left(s\right)\right)ds \end{array}$$

**Figure 4:**
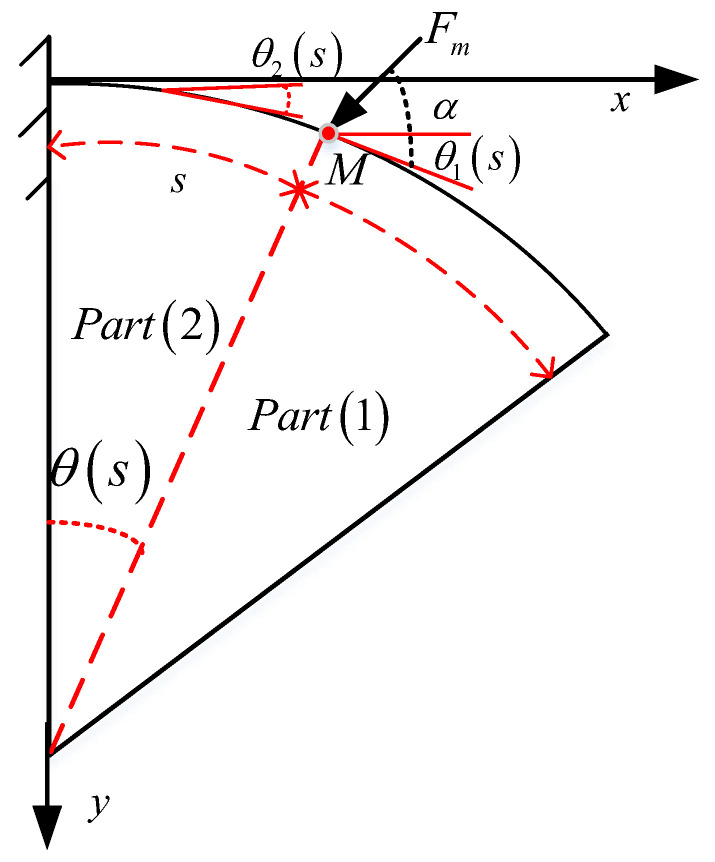
Diagram of the soft arm subjected to an external vertical force.

During interactions between the soft arm and its environment, an external force may interfere with any part of the soft arm. For instance, an external force may be applied to a specific position in the middle of the soft arm. As shown in Figure 4, F is applied to point M of the soft arm and α is the angle between the force and the soft arm tangent. According to Euler–Bernoulli’s theory, *F* affects only Part (2), while Part (1) maintains the constant curvature shape.

The deformation of Part (2) of the soft arm can be obtained by solving the following ordinary differential equation:

(10) $$EI\left(\frac{d^{2}\theta \left(s\right)}{ds^{2}}-\frac{dk_{0}}{ds}\right)-F_{m}\sin\left(\theta \left(s\right)+\alpha -\theta_{0}\left(s\right)\right)=0,\left(0\leq s\leq M\right).$$

The boundary conditions are as follows:

(11) $${\theta^{\prime }}_{2}\left(M\right)=-k,\theta_{2}\left(0\right)=0.$$

The following equation applies to Part (1) of the soft arm:

(12) $$\theta^{\prime }\left(s\right)=\frac{1}{R},\left(M\leq s\leq L\right).$$

The Cartesian coordinates of the beam axis are then given by

(13) $$\begin{array}{l} x\left(s\right)=\left\{ \begin{array}{l} \int_{0}^{M}\cos\left(\theta_{2}\right)ds+\int_{M}^{s}\cos\left(\theta_{1}\right)ds\left(M\leq s\leq L\right)\\ \int_{0}^{s}\cos\left(\theta_{2}\right)ds\left(0\leq s\leq M\right) \end{array}\right.\\ y\left(s\right)=\left\{ \begin{array}{l} \int_{0}^{M}\sin\left(\theta_{2}\right)ds+\int_{M}^{s}\sin\left(\theta_{1}\right)ds\left(M\leq s\leq L\right)\\ \int_{0}^{s}\sin\left(\theta_{2}\right)ds\left(0\leq s\leq M\right) \end{array}\right. \end{array}$$

## 3. Experimental Platform Construction and Preliminary Model Verification Preparations

### 3.1. Experimental Platform

**Figure 5:**
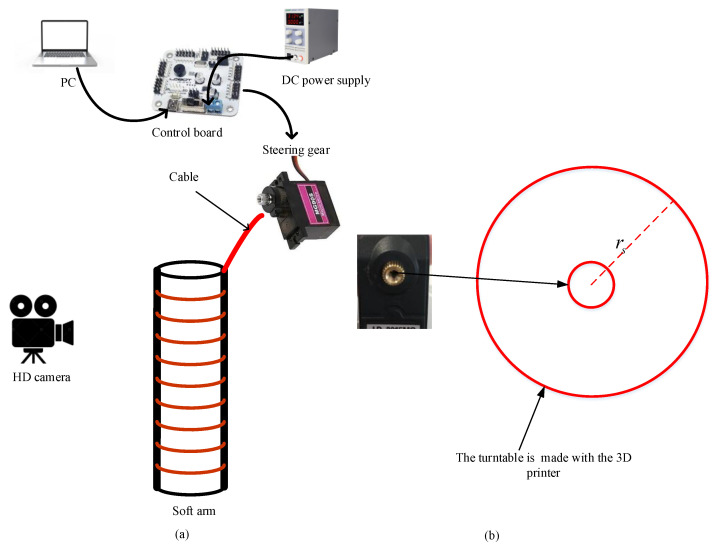
(**a**) Diagram of cable drive soft arm; (**b**) Turntable made by the 3D printer used to fix the cable on the steering gear.

**Figure 6:**
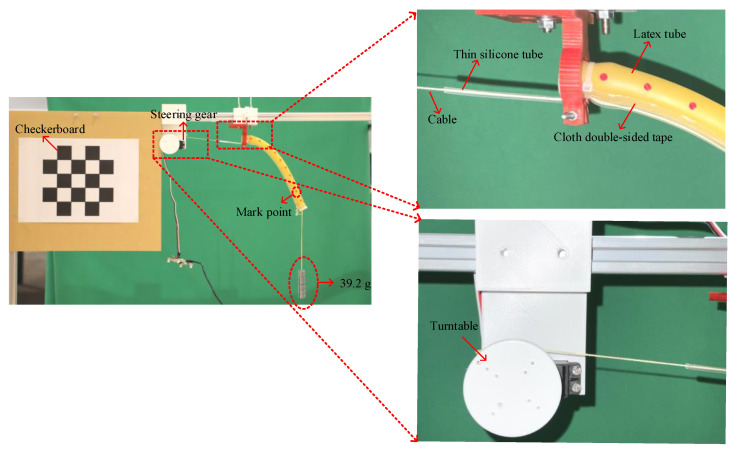
Experimental diagram of EI parameter fitting process.

As shown in Figure 5, the driving platform is mainly composed of a personal computer (PC), DC power supply, steering gear control board, steering gear, soft arm, and high-definition (HD) camera. The radius of the soft arm is 7.5 mm, its wall thickness is 2.5 mm, and its length is 235 mm. More detailed material parameters can be obtained from reference [10]. The body of the soft arm is made of a formed yellow latex tube. As shown in Figure 6, a thin silicone tube and the latex tube are fixed using a double-sided cloth tape. The thin silicone tube contains a cable and is fixed to the turntable, which is in turn fixed to the steering gear.

We do not add a curvature sensor to obtain the soft arm bending angle θ in practical applications. We drive the soft arm by connecting the steering gear and cable to solve this problem, as shown in Figure 5a. This paper uses steering gear rotation angles of 0–270 degrees, so the pulse width modulation (PWM) can be used to control the steering gear rotation angle. The PWM control interval is selected from 500 to 2500, and the position of the intermediate duty cycle is 1500. In other words, a 0-degree steering gear angle corresponds to 500, and a 270-degree steering gear angle corresponds to 2500. Therefore, the rotation angle of the steering gear can be calculated from the following equation:

(14) $$Steering\_Angle=\frac{PWM-500}{2500-500}\times 270,\left(500\leq PWM\leq 2500\right).$$

To facilitate the calculation of the cable’s length change, we fabricated a turntable using a 3D printer that can be fixed on the gear of the steering gearbox, as shown in Figure 5b. The radius of the disk is set to $r_{s}$ (20 mm). Using the rotation angle of the steering gear and the turntable radius, we can calculate the length change of the cable as:

(15) $$\Delta L=r_{s}\times Steering\_Angle.$$

The driving process of the soft arm is shown in Figure 6:

1. Control commands are sent to the steering gear control board through the steering gear computer software on the PC. The control commands are PWM.
2. After receiving the PWM data, the steering gear rotates and simultaneously drives the cable on the turntable to rotate them together.
3. The cable in the thin silicone tube is pulled by the rotation of the steering gear, causing the soft arm to bend.
4. The HD camera is used to record the soft arm shape in the model verification stage.

### 3.2. Preliminary Preparations for Model Verification

The preparatory work for model verification is divided into two parts. The first part is the fitting experiment of EI parameters, and the second part is the image processing of the soft arm. In Equations (4) and (5), we only need to know a single cable length variation to obtain the curvature. Therefore, in the verification experiment of the model proposed in this paper, we use a single cable to drive the soft arm.

Knowledge of the quantity EI is very important in our proposed model. Therefore, to accurately obtain the value of EI, this paper adopts a fitting method using multiple experiments. We suspend a 39.2-g load from the end of the soft arm, continuously change the shape of the soft arm using the steering gear drive and record it with an HD camera by identifying mark points, as shown in Figure 6.

PWM values of the steering gear are selected as 1415, 1485, 1535, 1600, 1660 to perform the fitting experiments. Through the analysis and comparison of experimental and model data, the best-fit EI value was 0.007 Pa⋅m^4^.

**Figure 7:**
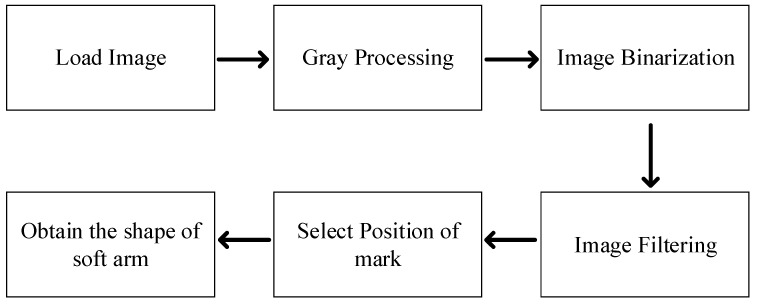
Image processing diagram.

The checkerboard is used to calculate the scale represented by each pixel of the camera, and the size of each checkerboard square is 3.5 cm × 3.5 cm. The image processing procedure is shown in Figure 7.

## 4. Results

We proposed two experimental schemes to verify the validity soft arm model:

(1) the end of the soft arm is loaded with different masses;
(2) the middle of the soft arm is loaded with a fixed mass, but the PWM value changes. The change in the PWM value corresponds to a change in the cable elongation.

Before starting the verification experiment, we first define a calculation method for the maximum error rate and use this formula to measure the model’s effectiveness:

(16) $$err\_rate=|\frac{MOD\_Data-EXP\_data}{L_{0}}|\times 100\%,$$

where MOD_Data represents the analytical model data, EXP_data represents the experimental data, and $L_{0}$ is the soft arm body length.

### 4.1. Model Verification of End-Suspended Load

**Figure 8:**
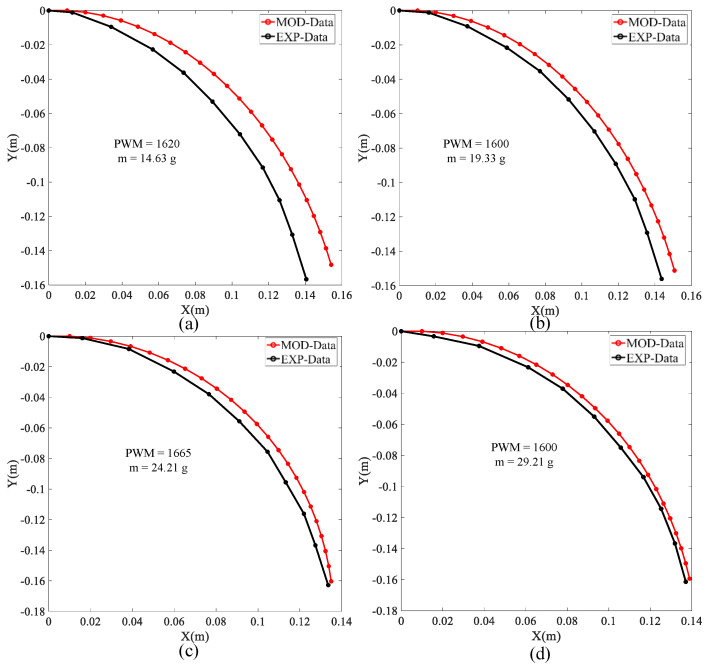
(**a**–**d**) shown the comparison of experimental and model analysis results for different mass loads suspended from the end of the soft arm.

**Figure 9:**
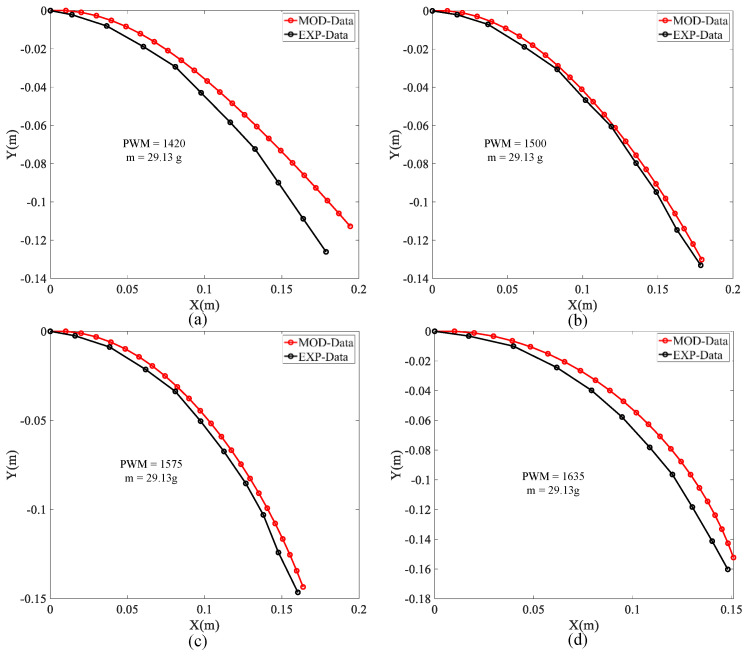
(**a**–**d**) shown the comparison of experimental and model analysis results for the same mass loads suspended from the middle of the soft arm.

The first verification experiment was conducted with loadings by different masses suspended from the end of the soft arm. Changing the loads and using different PWM values verified whether the proposed model was effective. As shown in Figure 8 and Figure 9, *MOD_Data* represents the simulation model data, and *EXP_Data* represents the data obtained by experimental measurement.

The masses suspended from the end of the soft arm are 14.63 g, 19.33 g, 24.21 g, and 29.21 g. The control range of PWM is between 1600 and 1665, and the initial servo PWM value is set to 1365.

As shown in Figure 8, the experimental and analytical model data become closer as the PWM and external loads increase. By comparing the experimental and model data in Figure 8, the maximum error rates for Figure 8a–d from Equation (16) are 6.96%, 3.51%, 3.97%, and 3.14%, respectively. Figure 8 also shows that the soft arm shape prediction model proposed in this article is still effective when changing the PWM and external load.

### 4.2. Model Verification for Mid-Suspended Loading

An experiment was also carried out to verify the model’s validity for a load suspended from the middle of the soft arm. We selected a 29.21-g mass to apply the external load, continuously increased the PWM value from 1420 to 1635, and recorded the soft arm shape with an HD camera. The external load was applied 12 cm from the fixed end of the soft arm (instead of fixing the load at the end of the soft arm).

Comparing the experimental and model data in Figure 9, the maximum error rates of Figure 9a–d calculated from Equation (16) are 8.75%, 2.85%, 3.22%, and 4.63%, respectively. For the condition with the external force applied to the middle of the soft arm, the maximum error rate estimated by the model is between 2.85% and 8.75%. Therefore, the model is effective and acceptable for predicting soft arm deformation.

## 5. Conclusions

This paper proposes a method that combines the constant curvature model with Euler–Bernoulli beam theory to effectively predict the soft arm shape, including the interference of external forces on the soft arm. We obtain the relationship between the cable length and curvature of the soft arm through the constant curvature model. The Euler–Bernoulli equation incorporates the influence of an external force on the soft arm shape into the model. Taking the obtained curvature as the initial condition for the Euler–Bernoulli beam ordinary differential equation, we can predict the soft arm deformation under the influence of an external force. We built a cable-driven soft arm platform and conducted a series of model verification experiments to verify the model’s effectiveness. In the experimental verification stage, we carried out model verification experiments for two cases, obtaining the following results:

(1) For the experimental verification with the external force applied to the end of the soft arm, the maximum error rate obtained by our model is between 3.14% and 6.96%;
(2) For the case with the external force applied to the middle of the soft arm, the maximum error rate obtained by our model is between 2.86% and 8.75%.

These experimental results show that our proposed model can be used to effectively predict the soft arm’s deformation.

In future work, we will continue to study the kinematic and dynamic modeling of the soft arm. In addition, we will also research ways to improve the output force of the soft arm. We hope that soft arms of the future will not only be soft but will also have increased power output.
